# Adverse outcomes of delayed intensive care unit

**DOI:** 10.5935/0103-507X.20200014

**Published:** 2020

**Authors:** Fernanda Ribeiro Quintino Santos, Maurício de Nassau Machado, Suzana Margareth Ajeje Lobo

**Affiliations:** 1 Hospital de Base, Faculdade de Medicina de São José do Rio Preto - São José do Rio Preto (SP), Brazil.

**Keywords:** Length of stay, Critical care, Hospital emergency service, Hospital mortality, Patient admission, Tempo de permanência, Cuidados críticos, Serviço hospitalar de emergência, Mortalidade hospitalar, Admissão do paciente

## Abstract

**Objective:**

To examine the impact of delayed transfer from the emergency room into the intensive care unit on the length of intensive care unit stay and death.

**Methods:**

This prospective, cohort study performed in a tertiary academic hospital obtained data from 1913 patients admitted to the emergency room with a documented request for admission into the intensive care unit. The patients admitted directly into the medical-surgical intensive care unit (n = 209) were categorized into tertiles according to their waiting time for intensive care unit admission (Group 1: < 637 min, Group 2: 637 to 1602 min, and Group 3: > 1602 min). Patients who stayed in the intensive care unit for longer than 3.2 days (median time of intensive care unit length of stay of all patients) were considered as having a prolonged intensive care unit stay.

**Results:**

A total of 6,176 patients were treated in the emergency room during the study period, among whom 1,913 (31%) required a bed in the intensive care unit. The median length of stay in the emergency room was 17 hours [9 to 33 hours]. Hospitalization for infection/sepsis was an independent predictor of prolonged intensive care unit stay (OR 2.75 95%CI 1.38 - 5.48, p = 0.004), but waiting time for intensive care unit admission was not. The mortality rate was higher in Group 3 (38%) than in Group 1 (31%) but the difference was not statistically significant.

**Conclusion:**

Delayed admission into the intensive care unit from the emergency room did not result in an increased intensive care unit stay or mortality.

## Abstract (pt)

**Objetivo:**

Verificar o impacto da transferência tardia do pronto-socorro para a unidade de terapia intensiva no tempo de permanência na unidade e na ocorrência de óbitos.

**Métodos:**

Este estudo de coorte prospectiva foi conduzido em um hospital acadêmico terciário, com obtenção dos dados de 1.913 pacientes admitidos ao pronto-socorro com solicitação documentada de admissão à unidade de terapia intensiva. Os pacientes admitidos diretamente para a unidade de terapia intensiva médico-cirúrgica (n = 209) foram categorizados em tercis segundo o tempo de espera para admissão à unidade de terapia intensiva (Grupo 1: menos de 637 minutos; Grupo 2: entre 637 e 1.602 minutos, e Grupo 3: acima de 1.602 minutos). Os pacientes que permaneceram na unidade de terapia intensiva por mais de 3,2 dias (tempo mediano de tempo de permanência na unidade de terapia intensiva para todos os pacientes) foram considerados como tempo prolongado de permanência na unidade de terapia intensiva.

**Resultados:**

Foram tratados no pronto-socorro durante o período do estudo 6.176 pacientes, dentre os quais 1.913 (31%) necessitaram de um leito na unidade de terapia intensiva. O tempo mediano de permanência no pronto-socorro foi de 17 horas (9 - 33 horas). Hospitalização por infecção/sepse foi preditor independente para tempo prolongado de permanência na unidade (RC: 2,75; IC95% 1,38 - 5,48, p = 0,004), porém o tempo de espera para admissão à unidade de terapia intensiva não. A taxa de mortalidade foi mais elevada no Grupo 3 (38%) do que no Grupo 1 (31%), porém a diferença não foi estatisticamente significante.

**Conclusão:**

A admissão tardia à unidade de terapia intensiva a partir do pronto-socorro não resultou em aumento do tempo de permanência ou da mortalidade na unidade de terapia intensiva.

## INTRODUCTION

Overcrowding of emergency rooms (ER) and intensive care units (ICU) is a reality, especially in the public health services of low-middle income countries. The population is aging, and thanks to advances in medicine, diseases such as acquired immunodeficiency syndrome and cancer have become chronic, increasing the demand for emergency services. Furthermore, complex diagnoses and treatments may currently be performed in the ER, such as blood transfusion and mechanical ventilation in patients with adult respiratory distress syndrome. These factors, along with higher ICU occupancy rates, have contributed to ER overcrowding and longer waiting times for transfer into the ICU.^(1,2)^

Emergency room patient overflow may lead to a delayed administration of antibiotics and analgesics, increased cases of pneumonia in intubated patients, delayed thrombolysis in patients with acute myocardial infarction, medication errors, and reduced quality of the services provided.^(3-8)^In addition, increased ICU occupancy was previously found to be associated with higher in-hospital mortality of patients with sepsis admitted via the emergency room.^(9)^Studies have shown that a delayed transfer of patients from the wards or ER units into the ICU is associated with worse outcomes in patients with sepsis and heterogeneous populations of critically ill patients,^(10-12)^and the risk of death nearly doubles in medical patients with delayed admissions when the hospital’s occupancy rate is higher than 90%.^(12)^

Critical patients who undergo prolonged hospital stays prior to intensive care may have a poorer outcome as a consequence of delayed monitoring and treatment administration. Epidemiological studies of the outcome of patients admitted in overcrowded ER, or those for whom an ICU bed was not promptly available, may draw attention to this matter and help in the planning of new strategies for emergency services for patients with these characteristics.

We hypothesized that longer waiting times for admissions into the ICU are associated with worse outcomes.

Our objective was to examine the impact of delayed transfer from the emergency room into the intensive care unit on the length of intensive care unit stay and death.

## METHODS

This prospective cohort study was performed between January 1 and December 31 of 2014 in the ER and medical-surgical ICU of a tertiary academic hospital (*Hospital de Base de São José do Rio Preto* - São Paulo, Brazil). The hospital had 471 beds for public care, 91 of which were ICU beds. This study was approved by the Ethics Committee (protocol number 866.419), which waived the requirement for informed consent. Patients were included if they were > 14 years or older and were admitted directly to the medical-surgical ICU (24 beds). During 2014, this ICU had an average occupancy rate of 92%. Patients who were discharged or died without ICU admission were excluded. Gynecology/obstetrics and pediatrics services were performed in an adjacent building and were not evaluated in this study.

Requests for ICU beds were made through the hospital’s electronic system with a summary of the clinical history, diagnosis, and priority classification adapted from the Society of Critical Care Medicine guidelines (Task Force of the American College of Critical Care Medicine); priority 1: critically ill unstable patients needing ICU treatment and monitoring, with significant likelihood of recovery; priority 2: stable patients who required intensive monitoring because of the possibility of decompensation; and priority 3: unstable patients with a low likelihood of recovery because of the severity of acute disease or because of comorbidities. ^(13)^Intensive care unit vacancies were managed by a physician who was continuously available through a 24-hour pager, along with the bed management service. The ER staff consisted of rotating residents, internal medicine and surgery certified physicians, and one board certified emergency physician on duty in the ER. Clinical and epidemiological data were obtained from the patients’ electronic medical records and included the Sequential Organ Failure Assessment (SOFA) score,^(14)^ dependency level,^(15)^ use of vasopressors, mechanical ventilation, need for renal replacement therapy (RRT), ICU and hospital length of stay and death.

Patients admitted to the ICU were divided into three groups according to the time spent in the ER while waiting for ICU admission: Group 1 < 637 min, Group 2 from 637 to 1,602 min, and Group 3 > 1,602 min. There is no consensus definition of prolonged ICU stay,^(16,17)^ so patients who stayed in the ICU for longer than 3.2 days (median time of hospitalization of all patients) were considered to have had a prolonged ICU stay. The outcomes evaluated were ICU and hospital length of stay and in-hospital death.

### Statistical analysis

Because of the non-Gaussian distribution, continuous variables were analyzed using the Mann-Whitney *U* test. Categorical variables were analyzed using either Pearson’s chi-squared test (w2) or Fisher’s exact test. Univariate and multivariate logistic regression (stepwise backward) were used to determine the independent predictors of prolonged ICU stay (above the median stay for the sample) and death. The independent variables used to adjust the model were ER length of stay (longer than 17 hours), age (years), sex (reference: male), admission due to neurological disease, admission due to cancer, admission due to infectious disease, history of arterial hypertension, level of dependency, chronic dialytic kidney disease and Glasgow Coma Scale on admission. Variables with a p value of < 0.25 were included in the multivariate model. Each variable entered in the multivariable model was proportional to at least 10 events in an attempt to avoid overfitting. The adjusted odds ratio (OR) and 95% confidence intervals (95%CIs) were calculated for the predictors. All statistical tests were performed using the IBM Statistical Package for Social Science (SPSS), version 22 Software (IBM Corporation, Armonk, NY, USA).

## RESULTS

**Figure 1:**
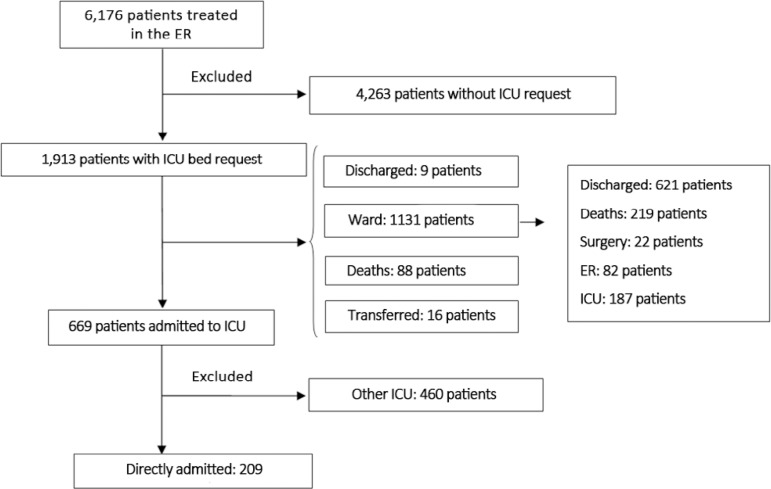
Flowchart of patient selection. ER - emergency room; ICU - intensive care unit.

During 2014, a total of 6176 patients were treated in the ER. Among those patients, 1,913 (31%) had a request for an ICU bed (Figure 1). Medical regulation via the Mobile Urgency Care Service (SAMU - *Serviço de Atendimento Móvel de Urgência*) and Central Health Services Regulation (CROSS - *Central de Regulação de Ofertas de Serviço de Saúde*) accounted for the referral of 1,033 patients (54%). The median age of the patients requiring an ICU bed was 63 years, and 60% were male. Medical emergencies accounted for 1,226 requests (64%), whereas surgical emergencies accounted for 687 (36%). The median length of the ER stay was 13 hours (interquartile 25% - 75%, 6 - 27 hours). During the study period, 1,045 patients were admitted to the ICU following transfer from the ER, wards, or surgical theater. The hospital’s overall mortality rate was 30% [22 - 34%].

A total of 209 patients (20%) were transferred from the ER into the medical-surgical ICU, among whom 129 (62%) were medical cases and 80 (48%) were surgical cases (Table 1). The median length of ER stay for those patients was 17 hours (range: 9 - 33 h). Approximately one-quarter of the patients required vasopressor support, and more than half (56%) had ventilator support initiated in the ER. The median length of ICU stay was 3.2 days (0.5 - 8 days, 25 - 75%) and that of the hospital stay was 13 days (6 - 24 days). The ICU mortality rate was 28%, and the hospital mortality rate was 34%.

**Table 1:** Clinical and epidemiological characteristics of patients included in the study

	All
Males	131 (63)
Age (years)	54 [34 - 64]
Chronic kidney dialysis	10 (5)
Neoplasia*	11 (5)
Arterial hypertension	73 (35)
Need for assistance†	31 (17)
Bedridden†	10 (5.5)
Glasgow Coma Scale^‡^	15 [12 - 15]
Diagnosis	
Cardiovascular diseases^§^	3 (1,5)
Neurological diseases^§^	62 (31)
Infectious diseases^§^	48 (24)
Pulmonary diseases^§^	10 (5)
Kidney diseases^§^	4 (2)
Other diseases^§^	74 (37)
Vasoactive drugs in ICU admission	
Noradrenaline	39 (19)
Dopamine^||^	7 (3)
Dobutamine^||^	6 (3)
Orotracheal intubation in ER	117 (56)
Orotracheal intubation in ICU	3 (1)
Length of stay	
Length of stay in hospital (hours)	17 [9 - 33]
Group 1	65 (31)
Group 2	71 (34)
Group 3	73 (35)
Length of stay in ICU (days)	3.2 [0.5 - 8]
Length of stay in hospital above median (3.2 days)	105 (50.2)
Total length of stay in hospital (days)	13 [6 - 24]
Mortality rate	72 (34)

ICU - intensive care unit; ER - emergency room. Missing data:

* 1 patient; missing data:

† 27 patients; excluded (sedation)

‡ 103 patients; missing data:

§ 8 patients; missing data:

¶ 4 patients; missing data:

|| 2 patients.

Results expressed as n (%) or median and interquartile (range 25% - 75%).

Table 2 shows the clinical characteristics, life support, and outcomes of all patients according to the length of ER stay. Younger patients were admitted into the ICU faster than older patients (Group 1, 48 years [median], 28 - 61 years, [25 - 75%]; Group 2, 52 years, 31 - 60 years; Group 3, 58 years, 44 - 72 years; p = 0.001). There were no differences in the length of ICU stay. The length of hospital stay increased significantly in Group 3 (18 days, 9 - 31 days) compared with Group 1 (10 days, 4 - 21 days) and Group 2 (11 days, 6.5 -20 days; p = 0.002), and the mortality rate was higher in Group 3 than in Group 1 (38% *versus* 31%; p = 0.639).

**Table 2:** Clinical and epidemiological characteristics of patients admitted to the intensive care unit stratified by their length of stay in the emergency room

	Group 1	Group 2	Group 3	p value
	(n = 65)	(n = 70)	(n = 71)	
Clinical admission	39 (60)	41 (58)	49 (67)	0.482
Male	42 (65)	43 (61)	46 (63)	0.885
Age (years)	48 [28 - 61]	52 [31 - 60]	58 [44 - 72]	0.001
Chronic kidney dialysis	2 (3)	4 (6)	4 (5.5)	0.739
Cancer	2 (3)	2 (3)	7 (10)	0.125
Arterial hypertension	21 (32)	23 (32)	29 (40)	0.567
Need for assistance	5 (9)	9 (14.5)	17 (26)	0.038
Bedridden	2 (4)	2 (3)	6 (9)	0.256
Glasgow coma scale	15 [13 - 15]	15 [12 - 15]	14 [12 - 15]	0.585
Diagnosis				
Cardiovascular diseases	1 (2)	1 (1,5)	1 (1)	0.996
Neurological diseases	18 (29)	17 (25)	27 (38)	0.245
Infectious diseases	12 (19)	19 (28)	17 (24)	0.461
Pulmonary diseases	4 (6)	4 (6)	2 (3)	0.579
Kidney diseases	1 (2)	1 (1.5)	2 (3)	0.824
Other diseases	27 (43)	25 (37)	22 (31)	0.362
Type of support at ICU admission				
Noradrenaline	11 (17.5)	13 (19)	15 (21)	0.877
Dopamine	3 (5)	3 (4)	1 (1)	0.506
Dobutamine	2 (3)	2 (3)	2 (3)	0.991
Orotracheal intubation in ER	36 (55)	41 (58)	40 (55)	0.932
Orotracheal intubation in ICU	0 (0,0)	1 (1)	2 (3)	0.402
ICU renal support	4 (6)	6 (9)	5 (7)	0.700
Outcomes				
Length of stay in ER (minutes)	375 [284 - 534]	973 [736 - 1229]	2693 [1930 - 4004.5]	< 0.001
Length of stay in ICU (days)	3 [0,8 - 10]	4 [0,7 - 8]	4 [0 - 9]	0.953
Prolonged hospital stay	31 (48)	37 (52)	37 (51)	0.872
Length of hospital stay (days)	10 [4 - 21]	11 [6,5 - 20]	18 [9 - 31]	0.002
ICU mortality rate	16 (25)	22 (31)	20 (27)	0,707
Hospital mortality rate	20 (31)	24 (34)	28 (38)	0,639

ICU - intensive care unit; ER - emergency room. Missing data were obtained for 1 patient in Group 2 and 2 patients in Group 3. Group 1: < 637 min; Group 2: 637 – 1,602 min; Group 3: > 1602 min. Results expressed as n (%) or median (25% - 75%).

Logistic regression analysis revealed that hospitalization for infection/sepsis was an independent predictor of prolonged ICU stay (OR 2.75 95%CI 1.38 - 5.48, p = 0.004), whereas age (OR 1.05 95%CI 1.02 - 1.09, p = 0.003) and the Glasgow Coma Scale (OR 0.82 95%CI 0.73 - 0.92, p = 0.001) were independent predictors of death in the ICU. These data are shown in table 3.

**Table 3:** Univariable and multivariable logistic regression analyses for predictors of prolonged intensive care unit stay and in-hospital death in mixed intensive care unit patients

	Univariate			Multivariate		
	OR	95%CI	p value	OR	95%CI	p value
Prolonged ICU stay						
Age (years)	1.00	0.99 - 1.01	0.954			
Sex (male)	0.86	0.49 - 1.51	0.604			
Neurological disease	0.68	0.37 - 1.24	0.206			
Infection/sepsis	2.75	1.38 - 5.48	0.004	2.75	1.38 - 5.48	0.004
Cancer	1.19	0.35 - 4.02	0.782			
Arterial hypertension	0.67	0.38 - 1.19	0.176			
Need for assistance	1.30	0.59 - 2.83	0.516			
Chronic renal dialysis	4.21	0.87 - 20.30	0.074			
Glasgow coma scale at admission	1.14	0.99 - 1.31	0.074			
ER length of stay (> 17 hours)	0.98	0.57 - 1.69	0.945			
In - hospital death						
Age (years)	1.04	1.02 - 1.06	< 0.001	1.05	1.02 - 1.09	0.003
Sex (male)	0.90	0.50 - 1.63	0.734			
Neurological disease	0.74	0.39 - 1.40	0.355			
Infection/sepsis	2.26	1.16 - 4.37	0.016			
Cancer	3.55	1.00 - 12.58	0.049			
Arterial hypertension	2.46	1.36 - 4.47	0.003			
Need for assistance	3.48	1.57 - 7.71	0.002			
Chronic renal dialysis	3.02	0.82 - 11.08	0.095			
Glasgow coma scale at admission	0.84	0.77 - 0.92	< 0.001	0.82	0.73 – 0.92	0.001
ER length of stay (> 17 hours)	1.20	0.68 - 2.13	0.527			

ICU - intensive care unit; OR - odds ratio; 95%CI - 95% confidence interval; ER - emergency room.

## DISCUSSION

This study gathered the following findings: (a) this hospital had a long ER stay; (b) longer ER stays were associated with increased hospitalization times; (c) younger patients were admitted faster into the ICU; and (d) independent predictors of prolonged ICU stay and death were identified.

The median length of ER stay among patients admitted to the ICU was 17 hours, which is longer than recommended. According to the Society of Critical Care Medicine, the transfer of a critical patient from the ER to the ICU should be performed within a maximum of 6 hours.^(13)^ In Australia, an acceptable length of ER stay is up to 8 hours, and in the United Kingdom, it is up to 4 hours.^(18)^ In our study, the main factor underlying the delayed transfer from the ER into the ICU was the ICU occupancy rate, which was higher than 90%. Other frequently related factors were bed unavailability in the wards, which limited ICU discharge, lack of bedding equipment, delayed bed release because of medical visits, bed cleaning, family visits, and intercurrences in the sector, among others.

Our study revealed a correlation between ER waiting time and hospital stay but not with ICU stay or death. Indeed, in our study, delayed ICU admission was not an independent predictor of prolonged ICU stay or death in the multivariate analysis. The length of hospital stay increased significantly in group 3 (18 days) compared with group 1 (10 days) and group 2 (11 days). We observed increases in death rates with increasing length of stay in the ER, although the difference was not statistically significant. Several authors have shown that, upon admission to an academic hospital’s ER, both the length of hospital stay and the mortality rate were higher in patients who remained longer in the ER.^(19)^ A study with 267 patients on mechanical ventilation in the ER showed that waiting longer than 1 hour was related to a longer ICU stay and longer need for ventilatory support.^(20)^ Critical patients who remain in the ER for six or more hours have an increased length of hospital stay and increased hospital and ICU mortality.^(21)^

In this study, mortality rates increased from 31% in Group 1 to 34% in Group 2 and 38% in Group 3, but because of the sample size, it was not possible to demonstrate statistical significance. A previous study in a public university hospital in Brazil found that ICU transfers were commonly delayed, with an average waiting time of 18 hours, which was associated with increased organ dysfunction. The same study revealed that mortality rates increased from 38% in the immediate admission group to 46% for waiting times longer than 12 hours and 57% for waiting times longer than 24 hours; the risk of death in the ICU increases by 1.5% for each 1 hour of waiting.^(10)^ Another study, which included 122 patients, showed that mortality increased from 14% in patients admitted immediately to 35% in patients admitted within 24 hours.^(11)^

Patients in Group 1 were significantly younger, demonstrating that, given the lack of resources, the health professionals of this hospital prioritize the admission of younger patients. As no risk score was used in the ER, we cannot say whether these patients were more severely ill. In the group that spent more time in the ER, the median age was higher. Forero et al.^(18)^ reported similar results, showing that older patients remained longer in the ER. We found that hospitalization due to infectious disease was an independent predictor of risk for prolonged ICU stay (above the group median). The best level of consciousness was an independent predictor of a better outcome. The prognostic importance of the level of consciousness at admission, as measured by the Glasgow Coma Scale score, is well known.^(22)^

Our data suggest that ER overcrowding, lack of ICU beds, and an increasing number of critically ill patients create an urgent need for new public health policies that enable the planning of preventive strategies. Brazil invests 4.3% of the gross national product in health, far below countries such as Canada (7.6%), the United States of America (8.3%) and Germany (8.6%).^(23)^ In particular, improved training of emergency physicians working with critically ill patients and a better design for health services are especially important. Such measures are necessary for patients to be admitted quickly to the ICU, where measures that impact the length of hospital stay and survival, such as goal-directed resuscitation, lung-protective ventilation strategies, early mobilization, daily interruption of sedation, and blood glucose control, are readily available.

The present study has few limitations, with the most important referring to the small sample size and the monocentric character of the study. However, our results provide important information, especially considering the lack of studies in this area, to alert the public health system about the human and financial losses that occur because of insufficient ICU beds.

## CONCLUSION

We conclude that longer stays in the emergency room did not result in increased intensive care unit stay or mortality but may be related to a longer overall hospital stay. Patients with infection/sepsis were at increased risk of longer intensive care unit stays.
